# Glycolytic reprogramming through PCK2 regulates tumor initiation of prostate cancer cells

**DOI:** 10.18632/oncotarget.18787

**Published:** 2017-06-28

**Authors:** Jiangsha Zhao, Jieran Li, Teresa W.M. Fan, Steven X. Hou

**Affiliations:** ^1^ The Basic Research Laboratory, National Cancer Institute, National Institutes of Health Frederick, Frederick, MD 21702, USA; ^2^ Graduate Center of Toxicology and Cancer Biology, Center for Environmental and Systems Biochemistry, and Markey Cancer Center, University of Kentucky, Lexington, KY 40536, USA

**Keywords:** cancer, glucose metabolism, phosphoenolpyruvate carboxykinase isoform 2, prostate, tumorigenicity

## Abstract

Tumor-initiating cells (TICs) play important roles in tumor progression and metastasis. Identifying the factors regulating TICs may open new avenues in cancer therapy. Here, we show that TIC-enriched prostate cancer cell clones use more glucose and secrete more lactate than TIC-low clones. We determined that elevated levels of phosphoenolpyruvate carboxykinase isoform 2 (PCK2) are critical for the metabolic switch and the maintenance of TICs in prostate cancer. Information from prostate cancer patient databases revealed that higher PCK2 levels correlated with more aggressive tumors and lower survival rates. PCK2 knockdown resulted in low TIC numbers, increased cytosolic acetyl-CoA and cellular protein acetylation. Our data suggest PCK2 promotes tumor initiation by lowering acetyl-CoA level through reducing the mitochondrial tricarboxylic acid (TCA) cycle. Thus, PCK2 is a potential therapeutic target for aggressive prostate tumors.

## INTRODUCTION

Emerging data suggest that a subpopulation of self-renewing and evolving tumor-initiating cells (TICs), popularly known as cancer stem cells (CSCs), may be responsible for tumor metastasis, and patient relapse and death [1]. Although the biological and clinical relevance of TICs/CSCs remains controversial, most solid tumors, including prostate cancer tumors, appear to follow the TIC/CSC model [2]. TICs are resistant to both radiation and chemotherapy in conventional treatments [3, 4]. Moreover, such treatments enrich TICs in tumors, endowing them with more aggressive characteristics. Unfortunately, there are no obvious molecular targets for TICs in rational drug design, because the molecular pathways underlying TIC maintenance are not well understood.

More than 75 years ago, Otto Warburg observed that tumor cells, like embryonic cells, preferentially use glycolysis to convert glucose carbon to lactate, even under aerobic conditions [5]. More recently, reports have shown that a single switch to the embryonic isoform of pyruvate kinase (PKM2) is necessary for the shift from OXPHOS to glycolysis in cancer cells and that this switch promotes tumorigenesis [6, 7]. The enhanced glycolysis in cancer cells can decrease reactive oxygen species (ROS), and promote the pentose phosphate pathway (PPP) and serine/glycine synthesis pathway, which are both linked to tumorigenesis [8–11].

In addition, yeast and many mammalian cells rely on acetyl-CoA for growth [12–15]. Acetyl-CoA is an essential building block for synthesis of fatty acids or sterols, or for the acetylation of histones on a set of more than 1,000 genes critical for cell growth [12]. In well-fed mammalian cells, the acetyl-CoA is primarily supplied by converting mitochondrial derived citrate into acetyl-CoA via ATP citrate lyase (ACLY) [16, 17]. In highly glycolytic or hypoxic tumors, glucose-derived pyruvate is preferentially shunted toward lactate instead of entering into the mitochondrial tricarboxylic acid (TCA) cycle for production of citrate and acetyl-CoA. It was recently reported that the nucleocytosolic acetyl-CoA synthetase enzyme, ACSS2, converts acetate into a key source of acetyl-CoA for tumors under such conditions [13, 14].

Many stem cells rely heavily on aerobic glycolysis instead of the TCA cycle to produce ATP. In addition, ROS production is relatively low due to low OXPHOS; stem cells are sensitive to ROS, and either die or differentiate under excessive ROS [18, 19]. TICs from different types of cancers exhibit higher glycolysis levels than non-TICs [20, 21]. Also similar to TICs, the metabolic switch from OXPHOS to aerobic glycolysis regulates the reprogramming of somatic differentiated cells into induced pluripotent stem cells (iPSCs) [18, 19].

The above findings suggest a connection between TICs and metabolic reprogramming; however, the significance of this connection is still unclear. Here we show that elevated levels of phosphoenolpyruvate carboxykinase isoform 2 (PCK2) are critical for the metabolic switch and maintenance of TICs in prostate cancer. TIC-enriched clones expressed a high level of PCK2, and PCK2 knockdown resulted in low numbers of TICs. In contrast, PKM2 knockdown resulted in high TICs. Prostate cancer patient database information revealed that higher levels of PCK2 expression were associated with more aggressive tumors and lower survival rates. PCK2 knockdown resulted in low TIC numbers, increased cytosolic acetyl-CoA and cellular protein acetylation. Our data suggest PCK2 regulates tumor initiation of prostate cancer cells by reducing the mitochondrial tricarboxylic acid (TCA) cycle activity and thereby production of citrate and acetyl-CoA. Our findings suggest that PCK2 is critical for the metabolic switch in tumor initiation, and that PCK2 is a potential therapeutic target for aggressive prostate tumors.

## RESULTS

### Isolation of TIC-enriched subclones of prostate cancer cell lines

**Figure 1:**
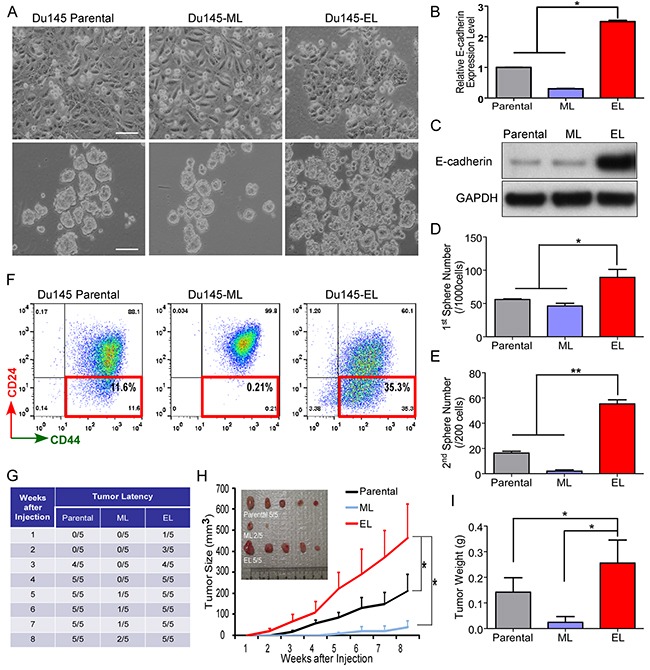
Isolation and characterization of a TIC-enriched single-cell clone from Du145 cells **(A)** Morphology of the Du145 parental cells, Du145-ML clone, and Du145-EL clone (upper), and the first spheres cultured from these cells (bottom). Scale bar, 100 μm. **(B and C)** E-cadherin expression level in the Du145 parental cells, Du145-ML clone, and Du145-EL clone detected by qPCR and immunoblotting. **(D and E)** Numbers of first- and second-generation spheres cultured from the Du145 parental cells, Du145-ML clone, and Du145-EL clone. **(F)** Representative flow cytometry results of CD44+/CD24- TICs in the Du145 parental cells, Du145-ML clone, and Du145-EL clone. **(G)** Latency of tumor growth in nude mice. **(H)** Tumor growth over time. Data are the mean ± SEM from five mice. **(I)** Tumor weight when the xenograft assay was terminated. Data are the mean ± SEM from five mice. ^*^: p < 0.05.

Heterogeneous subclones can be isolated from prostate cancer cell lines by culturing single-cell clones [22, 23]. Using this method, we isolated several subclones from two prostate cancer cell lines, Du145 and PC3/M. These clones could be stably subcultured (data not shown). Most of the subclones from Du145 could be divided into two major types based on their morphology: mesenchymal or epithelial-like (Figure 1A). We randomly picked one clone of each type for further analysis. Consistent with their morphologies, clone D18 (epithelial-like, hereafter Du145-EL) showed significantly higher E-cadherin expression than the parental cells or the mesenchymal-like clone B2 (hereafter Du145-ML) (Figure 1B and 1C). Although a similar morphological difference was not observed between the single-cell PC3/M subclones (Supplementary Figure 1A), they could also be divided into two types based on their E-cadherin expression (Supplementary Figure 1B), and we have used PC3/M-EL to designate the high E-cadherin expressers and PC3/M-ML to denote the low E-cadherin clones.

The cell status has been reported to be tightly related to TICs. Although many TICs have been found to have mesenchymal characteristics and epithelial-mesenchymal transition (EMT) can produce TICs in some cancer types [24, 25], there are also reports about TICs enriched in epithelial cell [24]. For example, cells with high expression of E-cadherin are reported enriched TICs in prostate cancer cells [26, 27]. Indeed, we found that the clones that highly expressed E-cadherin (Du145-EL and PC3/M-EL) contained many more CD44+/CD24- prostate cancer TICs [28, 29] than the E-cadherin-low clones (Du145-ML and PC3/M-ML) (Figures 1F and Supplementary Figure 1D). To further characterize the TICs, we examined the clones’ sphere-forming capacity [30]. Du145-EL cells had a strong sphere-forming ability, both in the first and second generations (Figures 1A, 1D, and 1E), compared to the parental and Du145-ML cells. A similar result was found for the clones derived from PC3/M: PC3/M-EL cells formed many more spheres than PC3/M-ML cells (Supplementary Figure 1A and S1C).

We next examined the tumor-initiating ability of these cells using *in vivo* xenograft assays. Du145-EL cells showed a much stronger tumor-initiating ability in nude mice than Du145-ML cells. Eight weeks after cell injection, 100% of the mice (5 out of 5) in the Du145-EL group grew tumors, while only 40% (2 out of 5) in the Du145-ML group did (Figure 1G), and the Du145-ML-derived tumors were significantly smaller (Figure 1H and 1I) and had a longer latency (Figure 1G) than the Du145-EL-derived ones.

Thus, the heterogeneous subclones were stable, and the EL clones, which were enriched in prostate cancer TICs, showed more aggressive characteristics than the ML clones.

### Enhanced glycolysis in TIC-enriched prostate cancer cells

**Figure 2:**
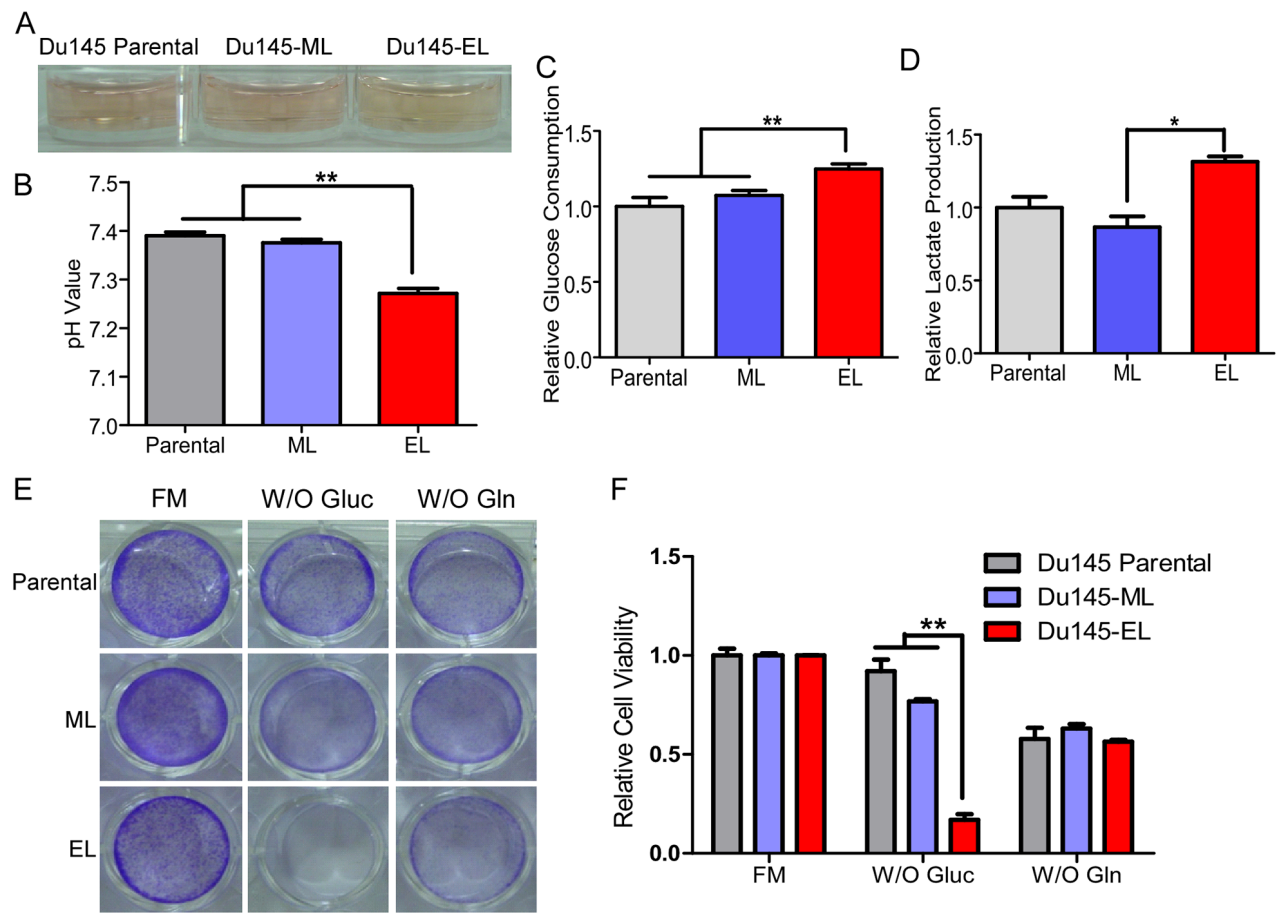
Enhanced glycolysis in TIC-enriched prostate cancer cells **(A)** Culture medium color of the Du145 parental cells, Du145-ML clone, and Du145-EL clone. **(B)** pH of culture medium from the Du145 parental cells, Du145-ML clone, and Du145-EL clone. **(C)** Glucose consumption in the Du145 parental cells, Du145-ML clone, and Du145-EL clone. **(D)** Lactate production by the Du145 parental cells, Du145-ML clone, and Du145-EL clone. **(E)** Cell viability detected by crystal violet staining after two days of glucose deprivation (W/O Gluc) or glutamine deprivation (W/O Gln). FM: full medium. **(F)** Quantification of cell viability after two days of glucose deprivation (W/O Gluc) or glutamine deprivation (W/O Gln). FM: full medium. ^*^: p < 0.05; ^**^: p < 0.01.

During daily cell culture, we noticed that the Du145-EL cell culture medium has a more acidic appearance than that of the Du145 parental and Du145-ML cell cultures (Figure 2A). A change in culture medium color indicates a change in its pH. We therefore cultured the same number of Du145 parental, Du145-EL, and Du145-ML cells in complete medium, and found that the medium from the Du145-EL cells had a lower pH than that from the other two (Figure 2B). This finding indicated that the cells had enhanced glycolysis, or increased glucose consumption and lactate production/secretion. Therefore, we measured the glucose consumption and lactate production, and found they were significantly elevated in the Du145-EL cells compared to the Du145 parental and Du145-ML cells (Figure 2C and 2D). The high glucose consumption in the Du145-EL cells led us to analyze the cells’ dependence on glucose for survival. Most Du145 parental and Du145-ML cells survived two days of glucose deprivation, most Du145-EL cells died under the same condition (Figure 2E and 2F), showing that the EL cells were intolerant of glucose deprivation. We also examined the response of the three cell groups to glutamine deprivation and found no significant differences between them (Figure 2E and 2F).

Although the PC3/M-EL and PC3/M-ML cells did not show significant differences in medium color or pH (data not shown), the PC3/M-EL cells showed greater glucose consumption and lactate production than the PC3/M-ML cells (Supplementary Figure 2A and 2B).

### High PCK2 expression in TIC-enriched prostate cancer cells

To understand the molecular basis for the glycolytic switch in the TIC-enriched cells, we first examined the PKM2 expression in our clones, because enhanced PKM2 expression is associated with the glycolytic switch in cancer cells [6]. However, we did not find elevated PKM2 expression in the EL clones (Supplementary Figure 2C), suggesting increased PKM2 expression was not responsible for their change in glucose metabolism.

**Figure 3:**
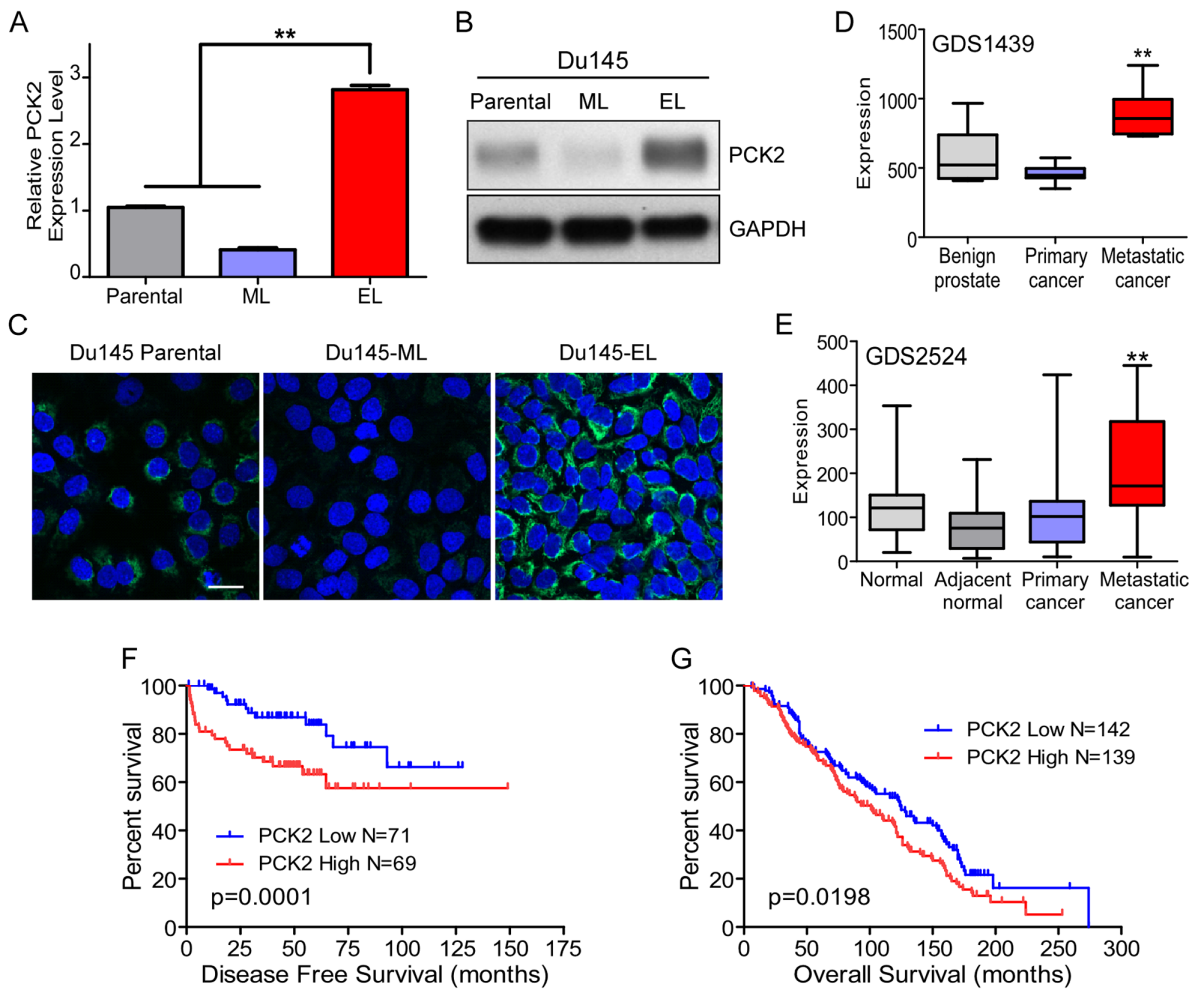
PCK2 is highly expressed in TIC-enriched prostate cancer cells and is a prognostic indicator for prostate cancer patients **(A and B)** PCK2 expression in the Du145 parental cells, Du145-ML clone, and Du145-EL clone by qPCR and immunoblotting. **(C)** PCK2 expression (green) in the Du145 parental cells, Du145-ML clone, and Du145-EL clone by immunofluorescence staining. Scale bar, 50 μm. **(D and E)** PCK2 expression in different subtypes of clinical prostate cancer samples. **(F and G)** Kaplan-Meier survival curves for prostate cancer patients grouped according to PCK2 expression. ^**^: p < 0.01.

To find genes responsible for this change, we compared the gene expression profiles of Du145-EL and Du145-ML cells using a microarray assay (GSE76470). Among the differentially expressed genes, the gene encoding PCK2, the mitochondrial form of phosphoenolpyruvate carboxykinase, was greatly elevated in the EL-clone cells. PCK2 catalyzes the conversion of oxaloacetate (OAA) to phosphoenolpyruvate (PEP) and is a key enzyme in the feeder reactions of carbon from the citric acid cycle to various biosynthetic processes, especially the synthesis of serine, glycerol, and nucleotides [31]. Using Q-PCR, Western blot, and immunofluoresence staining, we confirmed the elevated expression of PCK2 in Du145-EL cells (Figures 3A, 3B, and 3C). We also found that the PCK2 expression in PC3/M-derived clones was similarly elevated in the EL cells (Supplementary Figures 3A and 3B).

We next examined the PCK2 expression in clinical prostate cancer samples. Using published prostate cancer microarray datasets, we found a much higher PCK2 expression in metastatic prostate cancer samples compared to normal tissue or primary cancer tissue in two datasets (GDS1439 and GDS2524) (Figure 3D and 3E), indicating that PCK2 is highly expressed in aggressive prostate cancer. We then analyzed the correlation of PCK2 expression with patient survival in two independent cohorts (GSE20134 and GSE16560), and found that, in both cohorts, patients with high PCK2 expression showed a lower survival rate (Figure 3F and 3G).

### PCK2 is critical for the glycolytic switch in TIC-enriched cells

**Figure 4:**
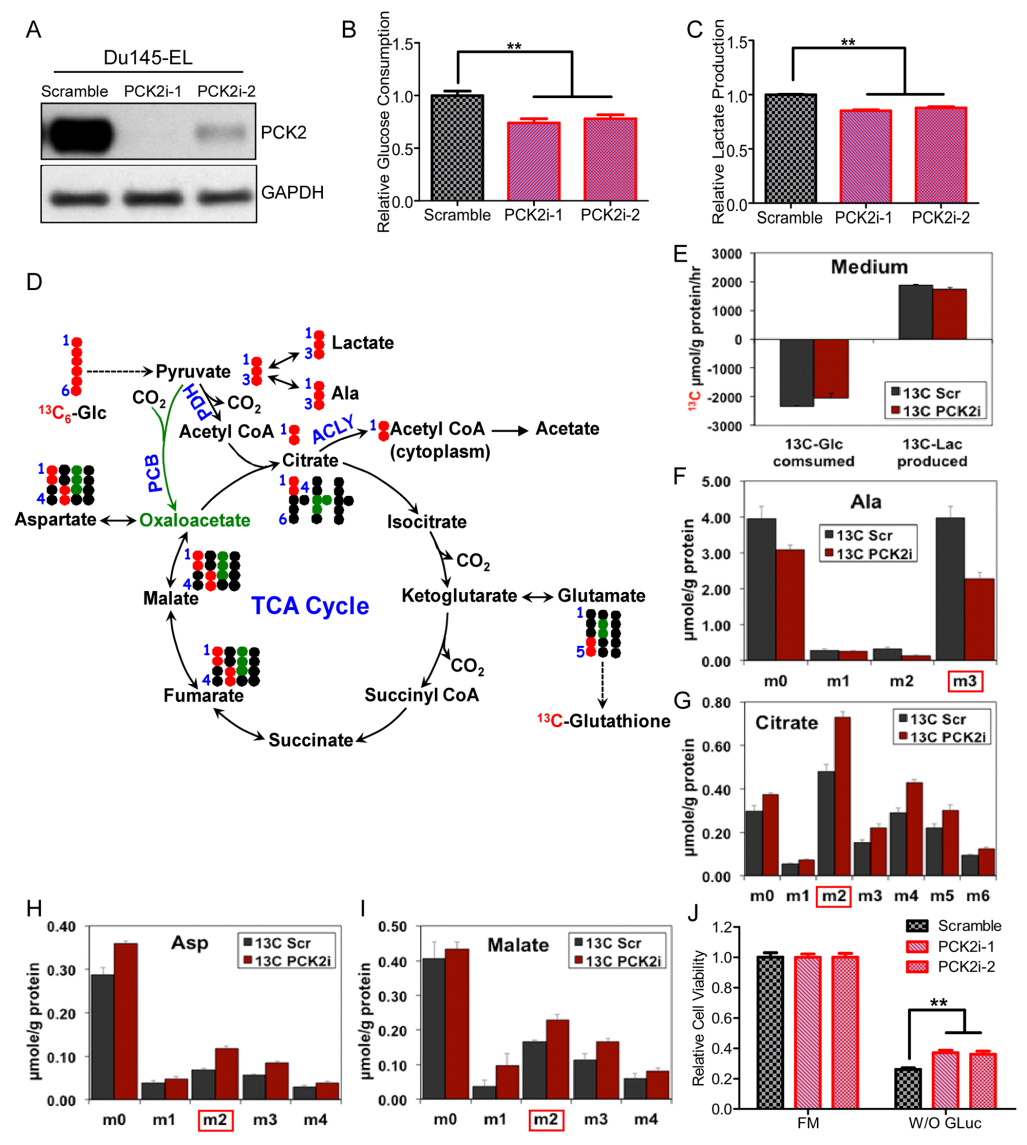
PCK2 is responsible for the reprogramming of glucose metabolism in TIC-enriched prostate cancer cells **(A)** PCK2 knockdown efficiency in Du145-EL cells, as detected by immunoblotting. **(B)** Glucose consumption in scramble control and PCK2-knockdown Du145-EL cells. **(C)** Lactate production by scramble control and PCK2-knockdown Du145-EL cells. **(D)** Diagram showing the expected ^13^C (red and green closed circle) labeling patterns of glycolytic and TCA cycle metabolites derived from ^13^C_6_-glucose. The TCA cycle reactions depicted represent PDH (red) or anaplerotic PCB-initiated (green) cycle activity for one cycle turn. PDH: pyruvate dehydrogenase; PCB: pyruvate carboxylase; ACLY: ATP-citrate lyase. **(E)** ^13^C_6_-Glucose uptake and ^13^C_3_-lactate production were determined by GC-MS analysis of the medium of Du145-EL scramble and PCKi-1 cells grown in ^13^C_6_-Glucose (mean ± SEM in triplicate). **(F to J)** ^13^C-labeled glycolytic and TCA cycle metabolites determined by GC-MS analysis. Data are the mean ± SEM of triplicate samples. Doubly and triply ^13^C labeled citrate are respectively unique markers of PDH and PCB-initiated TCA cycle activity (cf. diagram in D). M0 to m6 refer to mass isotopologues of TCA cycle metabolites with 0 to 6 ^13^C atoms. **(K)** Quantification of cell viability after two days of glucose deprivation (W/O Gluc). ^**^: p < 0.01.

Considering PCK2's regulation of OAA and PEP, we next examined whether PCK2 was responsible for the change in glucose metabolism in TIC-enriched EL cells. We used two shRNAs (PCK2i-1 and PCK2i-2) to knock down PCK2's expression in Du145-EL cells. PCK2i-1 almost completely blocked and PCK2i-2 dramatically reduced the PCK2 expression (Figure 4A). Consistent with the reported function of PCK2, knocking down PCK2 significantly reduced cellular PEP level (Supplementary Figure 4). We then measured the glucose consumption and lactate production in the PCK2-knockdown cells (PCK2i-1 or PCK2i-2), and found that both processes were decreased compared with control cells that were virally transfected with a scrambled sequence (Figure 4B and 4C). We also knocked down PCK2 in PC3/M-EL cells using the more efficient PCK2i-1 (Supplementary Figure 3C), and again found reduced glucose consumption and lactate production (Supplementary Figures 3D and 3E).

We next used the stable isotope-resolved metabolomics (SIRM) approach [32, 33] to analyze the metabolic fate of U-^13^C_6_-glucose in the Du145-EL scramble and PCK2i-1 cells. GS-MS was used to quantify ^13^C-labeled metabolites involved in glycolysis and the TCA cycle. Consistent with the results we obtained using unlabeled experiments, the SIRM results showed a trend of lower glucose consumption and lactate production in the PCK2-knockdown cells compared to the controls (Figures 4E). We also found significant increases in ^13^C2 (M2)-citrate, malate, aspartate (markers of the TCA cycle's first round),^13^C_3_-citrate (marker of pyruvate carboxylation) and ^13^C4 (M4)-citrate (marker of the TCA cycle's second round) (Figures 4F, 4G, 4H, and 4I). These results further indicated that PCK2 can remodel the glucose metabolism in TIC-enriched prostate cancer cells, by enhancing glycolysis and reducing the TCA cycle.

We then examined the sensitivity of PCK2-knockdown cells to two days of glucose deprivation, and found that the PCK2 knockdown partially rescued the cell death caused by glucose deprivation in both Du145-EL cells (Figure 4J) and PC3/M-EL cells (Supplementary Figure 3F).

### PCK2 and PKM2 differentially regulate TICs in prostate cancer cells

**Figure 5:**
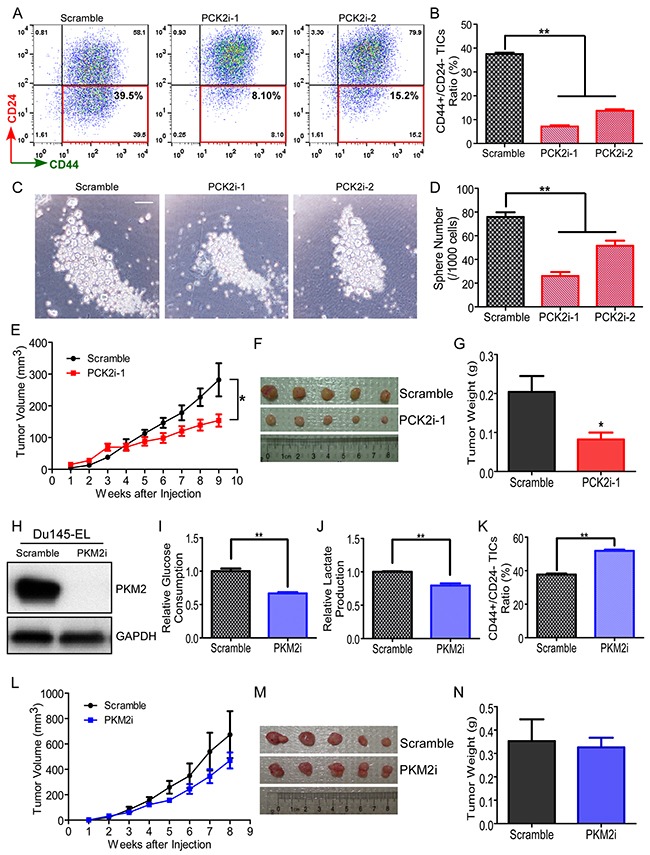
PCK2 and PKM2 differentially regulate TICs in prostate cancer cells **(A)** Representative flow cytometry results of CD44+/CD24- TICs in scramble control and PCK2-knockdown Du145-EL cells. **(B)** Quantification of CD44+/CD24- TICs in scramble control and PCK2-knockdown Du145-EL cells. **(C)** Representative sphere formation results from scramble control and PCK2-knockdown Du145-EL cells. Scale bar: 50 μm. **(D)** Quantification of spheres in scramble control and PCK2-knockdown u145-EL cells. **(E)** Du145-EL scramble and PCK2i-1 cells were injected into nude mice. Tumor volume was monitored weekly. Data are the mean ± SEM from five mice. **(F)** Tumors derived from Du145-EL scramble and PCK2i-1 cells. **(G)** Tumor weight when the xenograft assay was terminated. Data are the mean SEM from five mice. **(H)** PKM2 knockdown efficiency in Du145-EL cells, as detected by immunoblotting. **(I)** Glucose consumption in scramble control and PKM2-knockdown Du145-EL cells. **(J)** Lactate production by scramble control and PKM2-knockdown Du145-EL cells. **(K)** Quantification of CD44+/CD24- TICs in scramble control and PKM2-knockdown Du145-EL cells. **(L)** Du145-EL scramble and PKM2i cells were injected into nude mice. Tumor volume was monitored weekly. Data are the mean ± SEM from five mice. **(M)** Tumors derived from Du145-EL scramble and PKM2i cells. **(N)** Tumor weight when the xenograft assay was terminated. Data are the mean ± SEM from five mice. ^*^: p < 0.05; ^**^: p < 0.01.

To analyze whether PCK2 regulates the TIC characteristics of prostate cancer cells, we determined the number of TICs in PCK2-knockdown clones using flow cytometry. PCK2 knockdown using PCK2i-1 and PCK2i-2 reduced CD44+/CD24- TICs in the TIC-enriched Du145-EL cells by over 80% and 50%, respectively (Figure 5A and 5B). A similar result was observed in PC3/M-EL cells treated with PCK2i-1 (Supplementary Figure 5A and 5B), albeit less in extent for the reduction of CD44+/CD24- TICs. We also confirmed that the sphere-forming ability was significantly inhibited when the PCK2 expression was down-regulated in Du145-EL (Figure 5C and 5D) and PC3/M-EL (Figures S5C and S5D) cells. Consistent with our other findings, when the Du145-EL scramble control and PCK2i-1 cells were subcutaneously injected into nude mice and the xenograft growth was measured weekly, tumors derived from the PCK2-knockdown cells were significantly smaller than those derived from the scramble control (Figure 5E, 5F and 5G).

Although we did not find a significant difference in PKM2 expression between our ML and EL clones, because PKM2 is reported to have important roles of glycolytic control in cancer cells, we further analyzed its regulation of TICs in our system. We knocked down the PKM2 expression using a specific shRNA construct in both Du145-EL (Figure 5H) and PC3/M-EL cells (Supplementary Figure 5E). Consistent with previous reports [6], PKM2 knockdown significantly reduced glucose consumption and lactate production in both types of cells (Figures 5I, 5J, Supplementary Figure 5F, and 5G), and accumulated PEP (Supplementary Figure 4). We then analyzed the TICs by measuring the CD44+/CD24- subpopulation using flow cytometry. Surprisingly, in contrast to the effect of PCK2 knockdown, the down-regulation of PKM2 significantly increased the CD44+/CD24- TICs in both clones (Figures 5K, Supplementary Figure 5H, and 5I). This finding was inconsistent with previous reports showing that PKM2 knockdown can block the tumorigenicity of lung cancer and glioma cells [6, 34]. Interestingly, we also found that PKM2 knockdown in Du145-EL cells did not significantly affect the cells’ tumorigenicity in nude mice (Figure 5L, 5M, and 5N). These results suggest that PKM2 is not important for the maintenance of TICs in prostate cancer.

### PCK2 and PKM2 affect TICs by differentially regulating the ROS status

**Figure 6:**
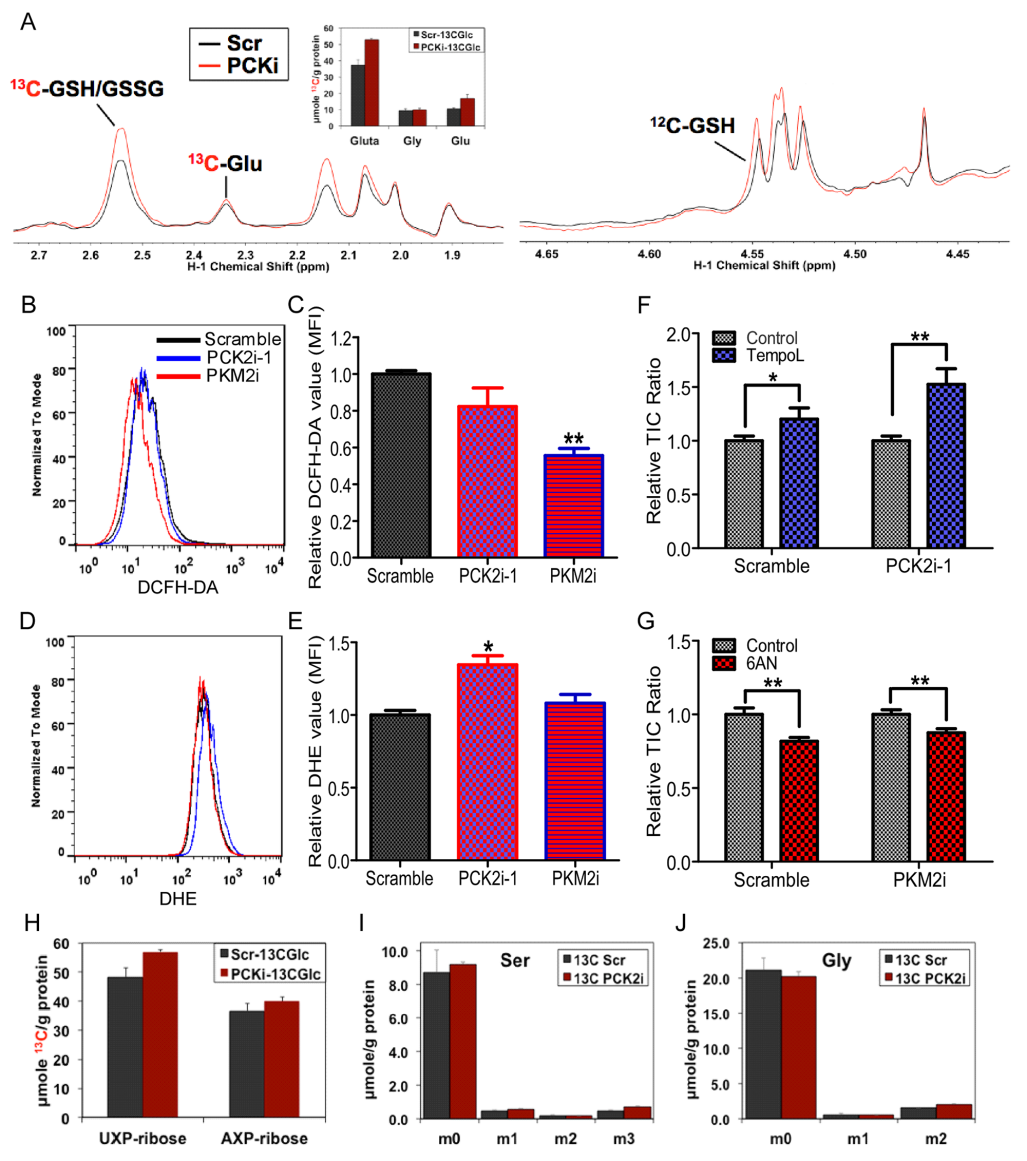
PCK2 and PKM2 differentially regulate cellular ROS **(A)** Representative HSQC NMR spectra showing the changes in ^13^C abundance (represented by the intensity of ^13^C-attached ^1^H peaks) of various assigned metabolites in the Du145-EL scramble (Scr, black) and PCK2i-1 (PCKi, red) cells. Also shown is the bar graph of average levels of ^13^C labeled glutathiones (Gluta, GSH+GSSG) and their precursors Gly and Glu; data are represented as mean ± SEM in triplicate. **(B)** Representative flow cytometry results of the H_2_O_2_ level detected by DCFH-DA staining in the scramble control, and PCK2- and PKM2-knockdown Du145-EL cells. **(C)** Quantification of the H_2_O_2_ level in the scramble control, and the PCK2- and PKM2-knockdown Du145-EL cells. MFI: mean fluorescence intensity, ^**^: p < 0.01 (compared to scramble control). **(D)** Representative flow cytometry results of the O2^·-^ level detected by DHE staining in the scramble control, and the PCK2- and PKM- knockdown Du145-EL cells. **(E)** Quantification of the O2^·-^ level in the scramble control, and the PCK2- and PKM2-knockdown Du145-EL cells. MFI: mean fluorescence intensity, ^*^: p < 0.05 (compared to scramble control). **(F)** Quantification of CD44+/CD24- TICs in the scramble control and PCK2-knockdown Du145-EL cells after 1 mM TempoL treatment for 2 days. **(G)** Quantification of CD44+/CD24- TICs in the scramble control and PKM2-knockdown Du145-EL cells after 10 μM 6-AN treatment for 2 days. (H) HSQC NMR uantification of ^13^C labeled ribose moiety of adenine nucleotides (AXP) and uracil nucleotides (UXP) in Du145-EL Scramble and PCK2i-1 cells, data are represented as mean ± SEM in triplicate. The ribosyl unit of AXP and UXP is derived from the pentose phosphate pathway (PPP). **(I and J)** GC-MS quantification of ^13^C labeled serine and glycine in Du145-EL Scramble and PCk2i-1 cells, data are represented as mean ± SEM in triplicate. M0 to m3 refer to mass isotopologues of Gly or Ser with 0 to 3 ^13^C atoms. ^*^: p < 0.05; ^**^: p < 0.01.

Our SIRM results also revealed an accumulation of reduced glutathione (GSH) and total ^13^C-labeled glutathione (GSH/GSSG) in the Du145-EL PCK2i-1 cells (Figures 6A). The latter suggest enhanced de novo glutathione synthesis by PCK knockdown. Glutathione is one of the major intracellular defenses against ROS [35], altered glutathione level and synthesis in Du145-EL PCK2i-1 cells indicated a change in ROS status. Two major intracellular ROS are hydrogen peroxide (H_2_O_2_) and superoxide (O2^·-^), both of which are important for regulating the growth, survival, and maintenance of TICs [36]. As glutathione primarily reacts with H_2_O_2_, we first analyzed the H_2_O_2_ level with a specific probe, 2', 7’–dichlorofluorescin diacetate (DCFH-DA). Surprisingly, we found only a slight, non-significant decrease in the DCFH-DA signal in Du145-EL PCK2i-1 cells (Figure 6B and 6C). Since GSH level also increased in Du145-EL PCK2i-1, it is possible that PCK suppressed cells have less need for GSH for H_2_O_2_ removal. As H_2_O_2_ is largely derived from O2^·-^, we next examined the intracellular O2^·-^ level with a specific superoxide detection probe, dihydroethiodium (DHE) and found that the O2^·-^ level was significantly higher in the PCK2-knockdown Du145-EL cells (Figure 6D and 6E).

An increase in ROS is reported to decrease the TICs in cancer cells [37]. To examine whether the increased O2^·-^, rather than H_2_O_2_, level in PCK2-knockdown cells was responsible for the TIC reduction, we treated the cells with two ROS scavengers, N-acetylcysteine (NAC) and TempoL. NAC acts as a synthetic precursor to glutathione synthesis, to enhance glutathione production and reduce the H_2_O_2_ level. NAC treatment did not rescue the TIC reduction phenotype in PCK2-knockdown cells (data not shown), which is consistent with the observed accumulation instead of depletion of GSH in these cells. However, treating the cells with TempoL, an O2^·-^-specific scavenger, significantly increased the proportion of TICs (TIC ratio) (Figure 6F). Furthermore, knocking down PCK2 in PC3/M-EL cells also induced a significant increase in O2^·-^, and TempoL rescued the TIC ratio in these cells (Supplementary Figure 6A, 6B, 6C, 6D, and 6E). However, increased H_2_O_2_ level was evident in PCK2 suppressed PC3/M-EL cells, unlike PCK2 knockdown Du145-EL cells. These results suggest that PCK2 can modulate TICs in TIC-enriched prostate cancer cells via regulating cellular ROS, particularly the O2^·-^ level.

Because PKM2 knockdown increased the TICs in prostate cancer clones, we also examined the ROS level in PKM2-knockdown cells. Unlike the PCK2 knockdown, PKM2 knockdown did not affect the O2^·-^ level in Du145-EL cells (Figure 6D and 6E). However, the H_2_O_2_ level was dramatically decreased in the PKM2i cells (Figure 6B and 6C). These results are consistent with a previous finding that inhibiting PKM2 activity can enhance the PPP and reduce the H_2_O_2_ level in lung cancer cells [11]. We therefore treated the cells with 6-aminonicotinamide (6-AN), a specific inhibitor for 6-phosphogluconate dehydrogenase, the first enzyme in the PPP. This treatment significantly decreased the proportion of TICs in the Du145-EL PKM2-knockdown cells (Figure 6G), indicating that the increase in TICs in PKM2-knockdown cells was partially due to the elimination of intracellular ROS through the PPP.

### PCK2 regulates TICs by modulating cellular acetylation

Two major pathways related to glucose metabolism that are important in regulating tumorigenicity are the PPP and the serine/glycine synthesis pathway [8, 9, 11]. However, our SIRM results indicated a slight enhancement, rather than attenuation of these two pathways in the Du145-EL PCK2i-1 cells (Figures 6H, 6I and 6J), indicating that some other mechanism must be involved in the PCK2-regulated TIC maintenance.

**Figure 7:**
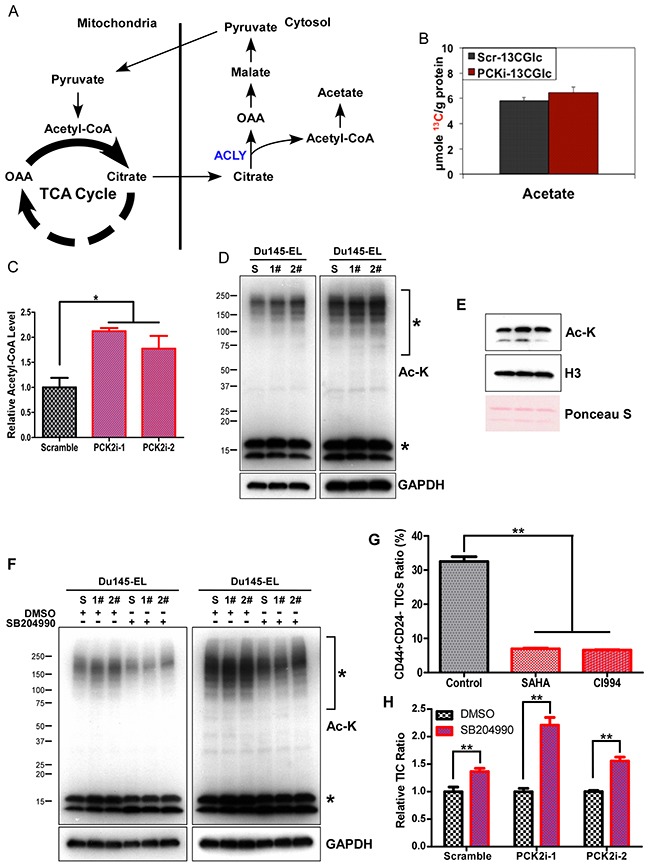
PCK2 regulates TIC maintenance by modulating protein acetylation via the citrate-pyruvate shuttle **(A)** Diagram showing the production of cytosolic acetyl-CoA by ACLY. **(B)** Quantification of ^13^C-acetate level in Du145-EL scramble and PCK2i-1 cells from HSQC NMR results, data are represented as mean ± SEM in triplicate. **(C)** Quantification of acetyl-CoA in Du145-EL scramble and PCK2-knockdown cells. ^*^: p < 0.05. **(D)** Cellular acetylation level detected by western blotting in cell lysates of scramble control and PCK2-knockdown Du145-EL. Short (left) and longer (right) exposures were used to show the differences. ^*^ indicates bands that were different between the scramble and PCK2-knockdown cells. **(E)** Histone acetylation detected by western blotting in acid-extracted histone samples from scramble control and PCK2-knockdown Du145-EL cell lysates. **(F)** Cells were treated with 100 μM SB204990 for 2 days, then the acetylation level was detected by western blotting in cell lysates. Short (left) and longer (right) exposures were used to show the differences. ^*^ indicates bands that were different between the scramble and PCK2-knockdown cells. **(G)** Quantification of CD44+/CD24- TICs in Du145-EL cells after 2 μM SAHA or 10 μM CI994 treatment for 2 days. (H) Quantification of CD44+/CD24- TICs in scramble control and PCK2-knockdown Du145-EL cells after 100 μM SB204990 treatment for 2 days. ^**^: p < 0.01.

When analyzing the SIRM results, we noticed a small increase of ^13^C-acetate (product of acetyl CoA) level in PCK2i-1 cells (Figure 7B), in addition to increased production of ^13^C-citrate (precursor to cytoplasmic acetyl CoA) (Figure 4D). These data point to an enhanced acetyl CoA production from citrate in the cytoplasm via the ATP citrate lyase (ACLY) action. Consistent with the SIRM results, the acetyl-CoA was also significantly increased in PCK2i cells (Figure 7C).

Given that acetyl-CoA is a major regulator of protein acetylation [12, 17], and protein acetylation regulates cancer cell proliferation, survival, and even TIC maintenance [38, 39], we examined protein acetylation in the PCK2-knockdown cells. Consistent with the increase in acetyl-CoA, the Du145-EL PCK2i-1 and PCK2i-2 cells showed enhanced acetylation in both whole-cell lysates and acid-extracted histones (Figure 7D and 7E). We also performed immunofluorescence staining, using an anti-acetylated lysine antibody, and found enhanced cytoplasmic staining of acetylated lysine in the Du145-EL PCK2i-1 cells (Supplementary Figure 7A). We then examined whether this enhanced acetylation was due to the increased acetyl-CoA production via ATP citrate lyase (ACLY) (Figure 7) in PCK2-knockdown cells. Since ACLY's production of cytoplasmic acetyl-CoA promotes protein acetylation [17], we treated the PCK2-knockdown cells with an ACLY-specific inhibitor, SB204990. This treatment dramatically reduced acetylation in the PCK2-knockdown cells (Figure 7F).

Since knocking down PCK2 reduced TICs in prostate cancer cells, we then asked whether this is related to cellular acetylation change. To mimic the enhanced acetylation in PCK2-knockdown cells, we treated Du145-EL cells with class I and class II histone deacetylase (HDAC) inhibitors, SAHA and CI994. SAHA and CI994 significantly reduced the TICs in Du145-EL cells (Figure 7G and 7B). We then examined whether inhibiting acetylation could rescue the TIC reduction in PCK2-knockdown cells. SB204990 treatment, which dramatically reduced the acetylation in PCK2 knockdown cells, also significantly increased the number of TICs in these cells (Figure 7H). Together, these results indicate that modulation of cellular acetyl CoA level and protein acetylation is an important factor in PCK2's ability to maintain TICs in prostate cancer cells.

## DISCUSSION

In this study, we grew up subclones from single cells of heterogeneous prostate cancer cell lines to obtain TIC-enriched and TIC-impoverished clones. We analyzed the clones’ CD44+/CD24- markers, *in vitro* sphere formation, and tumor formation in xenograft mice. We uncovered several important aspects of metabolic reprogramming and TIC maintenance in tumorigenesis. First, we found that selected TIC-enriched prostate cancer cell clones use more glucose and secrete more lactate than their TIC-low counterparts.Second,we determined that PCK2 is critical for the metabolic switch that generates and maintains TICs in prostate cancer. Third, we found that PCK2 and PKM2 have opposite roles in regulating TICs and ROS production. Fourth, PCK2 knockdown resulted in reduced TCA cycle and thereby lower citrate, cytosolic acetyl-CoA and cellular protein acetylation. Our data suggest that by shunting anion OAA as PEP from mitochondria to the cytosol PCK2 regulates tumor initiation of prostate cancer cells through reducing the TCA cycle, ROS level, and production of citrate and acetyl-CoA. Finally, using patient databases, we found that higher PCK2 expressions are associated with more aggressive tumors and lower survival rates in prostate cancer patients. Thus, PCK2 is a potential therapeutic target for aggressive prostate tumors.

### Compare PCK2 and PKM2 in regulating glycolytic reprogramming and TICs

PCK catalyzes the conversion of OAA to PEP [31]. It has two isoforms, a cytoplasmic form (PCK1, PEPCK-C) and a mitochondrial isoform (PCK2, PEPCK-M). PCK2 is a cataplerotic enzyme that can directly shunt excess citric acid cycle anion OAA as PEP from mitochondria to the cytosol. It was recently reported that PCK2 is highly expressed in different cancer cells and samples [40, 41]. A recent study also found that PCK2 can regulate TICs in melanoma cells [42]. However, PCK2 has been found to be downregulated in TICs in melanoma cells and repressed their tumorigenic ability. Opposite to those findings in melanoma, we found that PCK2 was upregulated in more aggressive prostate tumors and elevated PCK2 levels enriched TICs.

Recent studies have highlighted the importance of the biosynthetic and metabolic pathways of serine and glycine in cancer [43]. Serine and glycine are important components in the anabolic building blocks for the generation of glutathione, nucleotides, phospolipids, and other metabolites. PHGDH, the first enzyme of the de novo serine synthesis pathway, was found to be amplified in both breast cancer and melanoma [9, 10]. It was demonstrated that the pathways of aerobic glycolysis and the biosynthesis of serine and glycine are interconnected through PKM2 [44, 45]. Pyruvate kinase catalyzes the final step in glycolysis by transferring the phosphate from PEP to ADP, thereby generating pyruvate and ATP. Cancer cells selectively express the less active M2 isoform of pyruvate kinase (PKM2) [6]. Serine can bind to and activate PKM2. In a condition of serine deprivation, PKM2 activity was low, which would result in the accumulation of glycolytic metabolites (such as PEP) and the channeling of them into the serine biosynthesis to support cell proliferation [44, 45].

PCK2 directly shunts OAA as PEP from mitochondria to the cytosol; therefore both high PCK2 level and low PKM2 activity should result in accumulation of PEP and enhance serine biosynthesis to support cell proliferation. We found that the TIC-enriched clones express a high level of PCK2, and knocking down PCK2 significantly reduced CD44+/CD24- TICs in the TIC-enriched clones Du145-EL and PC3/M-EL; however, downregulation of PKM2 significantly increased CD44+/CD24- TICs in both clones. This is consistent with the potential functions of PKM2 and PCK2 in serine synthesis. Both the reduction of PKM2 activity and the elevation of PCK2 activity are support to channel more carbon through the TCA cycle to serine synthesis. However, our SIRM results indicated a slight enhancement, rather than attenuation of the PPP and the serine/glycine synthesis pathway in the Du145-EL PCK2i-1 cells (Figures 6H, 6I and 6J), indicating that the serine/glycine synthesis pathway may be not involved in the PCK2-regulated TIC maintenance. We further found that PCK2 knockdown resulted in reduced TCA cycle and thereby lower citrate, cytosolic acetyl-CoA and cellular protein acetylation. Therefore, PCK2 and PKM2 regulate TICs by distinct mechanisms. The reduction of PKM2 activity may channel more carbon through PEP to serine synthesis for increasing TICs, while the elevation of PCK2 activity will enrich TICs through reducing the TCA cycle, ROS level, and production of citrate and acetyl-CoA.

Consistent with our above observation, it has been noted that pluripotent stem cells primarily utilize glycolysis for their energy supply, while the differentiated cells rely on OXPHOS [18, 19]. Mitochondria, the central organelles in most cells, are the main sites for converting the final metabolites of carbohydrate, and lipid and amino acid to ATP and reactive oxygen species (ROS) through the OXPHOS processes by consuming O_2_ in the TCA cycle. However, the mitochondria in human and mouse embryonic stem cells are underdeveloped (a small number of rounded and non-fused cristae), while mitochondria in haematopoietic stem cells (HSCs) are relatively inactive. But after bone marrow damage, HSCs undergo rapid differentiation, and robust mitochondrial metabolism supplies the energy and ROS for this transition. In addition, many stem cells, including HSCs and mesenchymal stem cells reside in a hypoxic niche *in vivo* and are quiescent. They are generally sensitive to ROS. Excessive ROS induces either death or differentiation of stem cells. A lower level of ROS is critical for self-renewal of HSCs and CSCs in some human and murine breast tumors [37]. Together, these data suggest that stem cell metabolism can be reprogrammed on the basis of functional demands. Self-renewing stem cells rely mainly on aerobic glycolysis to maintain their quiescent state to ensure life-long tissue renewal capacity, but they have to rapidly switch to mitochondrial OXPHOS to meet the robust energy demands associated with differentiation [18, 19].

### PCK2 maintains TICs by reducing cellular acetyl-coA

In both yeast and many mammalian cells, elevated levels of acetyl-CoA are associated with cell growth [12–15]. The increased acetyl-CoA induces acetylation of histones on a set of more than 1,000 genes important for ribosome biogenesis, protein translation, and amino acid biosynthesis. Our metabolic characterization of PCK2 knockdown in prostate cancer cells found that knocking down PCK2 resulted in acetyl-CoA accumulation, increased protein acetylation, and dramatically reduced the proportion of TICs. Inhibiting acetylation with an ATP citrate lyase (ACLY)-specific inhibitor, SB204990, dramatically reduced acetylation and increased the number of TICs in the PCK2-knockdown cells (Figure 7). Our data suggest that PCK2 promotes tumor initiation through lowering acetyl-CoA level by shunting anion OAA as PEP from mitochondria to the cytosol and thereby reducing the TCA cycle.

There are two possible explanations for the appearing inconsistence between the published information and our result. First, decreased protein acetylation has been reported in some cancer cells, and increasing protein acetylation by inhibiting deacetylases such as histone deacetylases (HDACs) and NAD^+^-dependent sirtuin deacetylases (SIRTs), can inhibit certain cancer cells and CSC/TIC growth [46, 47]. Second, TICs are resistant to both radiation and chemotherapy (mostly targeting growing cells) in conventional treatments [3, 4, 48], such treatments enrich TICs in tumors and endowing them with more aggressive characteristics. TICs are relatively quiescent cells; poor conditions for growth (such as low acteyl-CoA) may favor their enrichment. Our results suggest that targeting tumors by lowering acetyl-CoA procedures may have the risk to enrich TICs.

### PCK2 is a potential therapeutic target for aggressive tumors

In the past few years, metabolic reprogramming has been rediscovered as a driving force of tumorigenesis and used as a new hallmark for cancer [49]. This finding has raised the new and exciting expectation that targeting metabolic enzymes, such as PKM2, may offer unique opportunities in cancer treatment [50]. Most tumor cells express relatively less active PKM2 dimers, and the lower PKM2 activity promotes the accumulation of upstream glycolytic intermediates, which are channeled into serine biosynthesis for cell proliferation [44, 45, 50]. Some small molecular activators of PKM2 are currently under development to suppress tumor growth by increasing pyruvate kinase activity [51].

However, recent findings from PKM2-knockout mice suggest that there is a differential requirement for pyruvate kinase activity among tumor cell populations [50]. Increased pyruvate kinase activity may favor the survival of some cancer cells, which complicates therapeutic strategies that target PKM2. Our study suggests that inhibiting PCK2 may overcome the shortcomings of targeting PKM2 and may be therapeutically valuable for cancers with elevated PCK2 expression. Elevated PCK2 has been reported in lung cancer cell lines, non-small cell lung cancer samples, and other types of cancer cells [40, 41]. In prostate cancer patients, high PCK2 is detected in more aggressive tumors, and patients with high PCK2 expression have lower survival rates. These findings indicate that PCK2 may play an important role in prostate cancer progression. Notably, we found that knocking down PCK2 could significantly reduce the proportion of TICs in prostate cancer cells, indicating that PCK2 is a potential target for therapies against TICs.

## MATERIALS AND METHODS

### Cell lines and single-cell clones’ isolation

Human prostate cancer cell lines Du145 and PC3/M (kindly provided by the DCTD Tumor Repository of NCI at Frederick, MD) were cultured in RPMI1640 supplemented with 10% fetal bovine serum and 100 units/ml penicillin/streptomycin, at 37°C in a humidified atmosphere containing 5% CO_2_.

Single-cell clones were isolated as previously reported [22, 23]. Briefly, cells were suspended in full culture medium at ~5 cells/ml, and then dispensed into 96-well culture plates at 200 μl/well. Each well was carefully checked under a phase-contrast microscope after plating. Wells containing just one cell were marked and checked daily. Holoclones that grew from the marked wells were sub-cultured and used for further analysis.

### RNA isolation and real-time PCR

Total RNA was extracted from cells using the RNeasy® Mini Kit (Qiagen), according to the manufacturer's instructions. Using a reverse transcription kit (Promega), 1 μg RNA from each sample was processed directly to cDNA. Amplification was performed in a 15-μL reaction system using SYBR^®^ Advantage^®^ qPCR Premix (Clotech). All of the reactions were performed in triplicate in a Realplex2 system (Eppendorf). The relative gene expression level was quantified as described previously [52].

The sequence of each primer was as follows:

Actin:

F: 5’- GATCATTGCTCCTCCTGAGC -3’

R: 5’- ACTCCTGCTTGCTGATCCAC -3’

E-Cadherin:

F: 5’- ACCAGAATAAAGACCAAGTGACCA -3’

R: 5’- AGCAAGAGCAGCAGAATCAGAAT -3’

PCK2:

F: 5’- CATCCGAAAGCTCCCCAAGT -3’

R: 5’- GCAGCCTGGAAACCTCTCAT -3’

### Gene microarray

Gene microarray assay was performed in Advanced Technology Research Facility at the NCI at Frederick, Frederick, MD, USA. Briefly, total RNA was extracted from DU145-ML and DU145-EL cells using the RNeasy® Mini Kit (Qiagen), according to the manufacturer's instructions. Qualified RNA was hybridized on Affymetrix human gene ST 1.0 microarrays. After scanning and normalization, the microarray data were analyzed by Partek Genomics Suite 6.6.

### Immunoblotting and immunofluoresence staining

Cells were washed twice with PBS and lysed in RIPA buffer. Protein concentrations were quantified using the Bradford reagent (Bio-Rad) according to the manufacturer's instructions. Samples with equal amounts of protein were separated by 4%–15% SDS-PAGE, then transferred to an Immobilon transfer membrane (Millipore) and immunoblotted with specific antibodies against E-cadherin, PCK2, PKM2, and Acetylated-Lysine (Ac-K^2^-100, #9814) from Cell Signaling, and GAPDH from Thermo Scientific. All of the immunoblots were visualized by enhanced chemiluminescene (Bio-Rad).

Immunofluoresence staining was performed to detect PCK2 expression and protein acetylation. Cells were plated in a 24-well multiwell glass-bottomed culture plate (MatTek), fixed in 4% formaldehyde in PBS for 15 min at room temperature, and then washed three times with PBS. After being blocked with PBS containing 5% goat serum and 0.3% Triton X-100 at room temperature for 1 h, the cells were incubated overnight with anti-PCK2 primary antibody (1:100, Cell Signaling) or anti- acetylated-Lysine (1:200, Cell Signaling #9441) at 4°C. The secondary antibody, Alexa Fluor 488-conjugated donkey anti-rabbit IgG (Molecular Probes) was used at a 1:1000 dilution. Fluorescence was monitored by an inverted confocal laser microscope (Carl Zeiss).

### Flow cytometry

Cell-surface markers and cellular ROS were analyzed by flow cytometry. To detect cell-surface markers, cells were dissociated by 0.05% trypsin-EDTA and centrifuged. The cells were resuspended in PBS containing 2% FBS and fluorescence-conjugated antibodies, FITC-CD44 (clone G44-26, BD Biosciences) and PE-CD24 or APC-CD24 (clone ML5, Biolegend), and incubated on ice for 30 min. After three washes in PBS containing 2% FBS, the cells were resuspended in PBS containing 2% FBS and analyzed by FACSCalibur (BD Biosciences).

To detect cellular ROS, cells were incubated with 5 ng/ml DCFH-DA (Santa Cruz) or 10 μM DHE (Life Technologies) at 37°C for 30 min. They were then dissociated by 0.05% trypsin-EDTA and centrifuged. After being washed with PBS containing 2% FBS, the cells were resuspended in PBS containing 2% FBS and analyzed by FACSCalibur (BD Biosciences).

### Sphere formation assay

Cells were cultured in a Corning® Costar® Ultra-Low attachment 24-well plate (Sigma-Aldrich) in sphere culture medium DMEM/F12 (1:1) with B27 (Invitrogen) and 20 ng/ml EGF (Invitrogen). For Du145 and its derived clones, new medium was added every three days. For PC3/M-derived clones, the cells were cultured in the sphere culture medium for three days, then sphere culture medium with 0.5% FBS was added every three days. The spheres were counted after 10 days in culture.

### Glucose, lactate, PEP and acetyl-coA detection

The concentration of glucose and lactate in the cell culture medium and of PEP and acetyl-CoA in cell lysate was detected by a glucose assay kit (Eton Bioscience), L-lactate assay kit I (Eton Bioscience), PEP Fluorometric Assay Kit (Cayman) and the PicoProbe™ Acetyl-CoA Fluorometric Assay Kit (Biovision), respectively. The assays were performed as instructed by the manufacturer.

### shRNA knockdown of PCK2 and PKM2

The PCK2 shRNA constructs (TRCN0000052666 and TRCN0000052667) were purchased from MISSION shRNA at Sigma-Aldrich. A PKM2 shRNA construct (plasmid: 42516) and a scramble control construct (plasmid: 1864) were purchased from Addgene. The shRNA constructs were co-transfected with three lentiviral packaging plasmids into 293T cells. The culture medium containing lentivirus was harvested two days after transfection. Cells were infected with the harvested medium (filtered with 0.45 μm filter) and selected with 1 μg/ml puromycin (Invivogen).

### In vivo tumorigenicity assay

Cancer cells were injected into the flank of six-week-old male nude mice. The cell amounts were as follows: 1 million/site for Du145 parental, Du145-ML, and Du145-EL cells; 0.4 million/site for scramble control and PCK2-knockdown (PCK2i-1) Du145-EL cells; 1 million/site for scramble control and PKM2-knockdown Du145-EL cells. Tumors were measured weekly with a caliper and calculated using the formula: volume = 0.5 × length × width^2^. The results are presented as the mean ± SE. All animals used in this research project were cared for and treated humanely according to the following policies: the U.S. Public Health Service Policy on Humane Care and Use of Laboratory Animals (2000); the Guide for the Care and Use of Laboratory Animals (1996); and the U.S. Government Principles for Utilization and Care of Vertebrate Animals Used in Testing, Research, and Training (1985). All NCI at Frederick animal facilities and the animal program are accredited by the Association for Assessment and Accreditation of Laboratory Animal Care International.

### Stable isotope-resolved metabolomic (SIRM) experiment with ^13^C_6_-glucose tracer

Du145-EL scramble (Scr) and PCK2i-1 cells were cultured in the RPMI1640 medium with 0.2 % unlabeled or ^13^C_6_-glucose for 24 hrs, as described previously [53]. Acetonitrile/water/chloroform (2/1.5/1, v/v) partitioning method was used to extract polar metabolites from cell and media. The extracts were subjected to NMR and GC-MS analysis as previously described [32].

### Statistical analysis

Statistical analysis was performed using SPSS 13.0 for Windows. The data were presented as mean values ± standard deviation, except those from the *in vivo* tumorigenicity assay. Statistically significant differences were determined by Student's t-test and one-way ANOVA, where appropriate, and defined as P < 0.05.

## SUPPLEMENTARY MATERIALS FIGURES


