# Supplementary material for: Glycolytic reprogramming through PCK2 regulates tumor initiation of prostate cancer cells
Source: Oncotarget. 2017 Jun 28;8(48):83602–18. doi: 10.18632/oncotarget.18787 (PMC5663539; doi:10.18632/oncotarget.18787)
Supplement: Supplementary file 1 [file oncotarget-08-83602-s001.pdf]

Glycolytic reprogramming through PCK2 regulates tumor Initiation of Prostate Cancer Cells

SUPPLEMENTARY MATERIALS

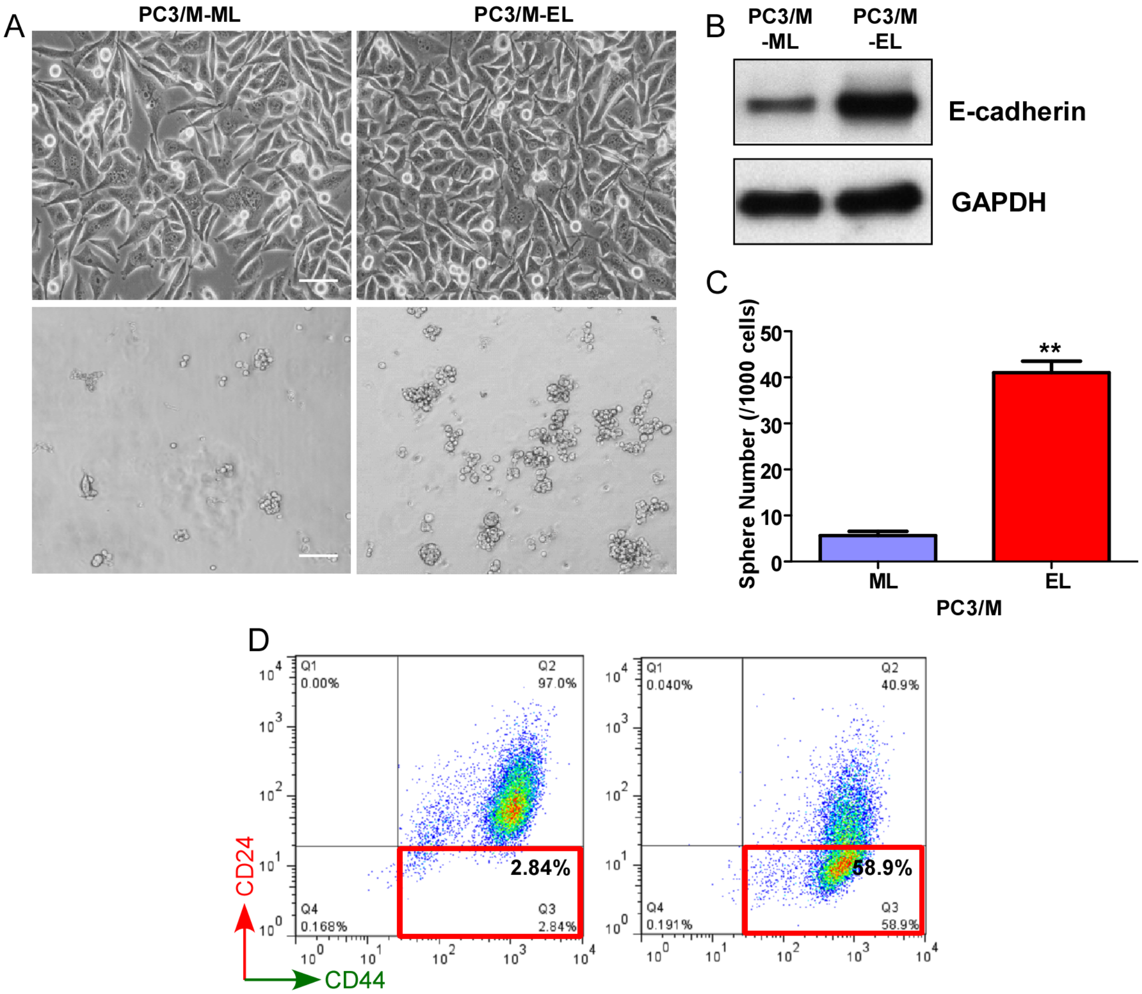

Supplementary Figure 1: Isolation and characterization of a TIC-enriched single-cell clone from PC3/M cells (A) Morphology of the PC3/M-ML clone and PC3/M-EL clone (upper), and the first spheres cultured from these cells (bottom). Scale bar, 100 µm. **(B)** E-cadherin expression level in the PC3/M-ML clone and PC3/M-EL clone, detected by immunoblotting. **(C)** Numbers of spheres cultured from the PC3/M-ML and PC3/M-EL clones. **(D)** Representative flow cytometry results of CD44+/CD24- TICs in the PC3/M-ML and PC3/M-EL clones. *: $p < 0.05$; **: $p < 0.01$.

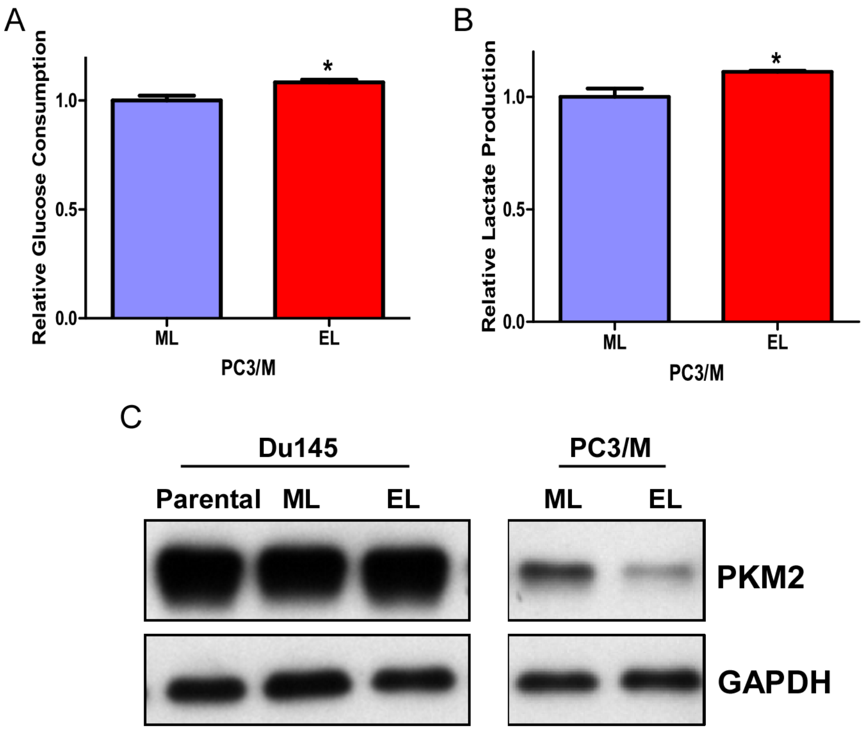

Supplementary Figure 2: Enhanced glycolysis and PKM2 detection in the PC3/M-EL clone (A) Glucose consumption in the PC3/M-ML and PC3/M-EL clones. (B) Lactate production by the PC3/M-ML and PC3/M-EL clones. **(C)** PKM2 expression in Du145-derived and PC3/M-derived clones detected by mmunoblotting. *: $p < 0.05$.

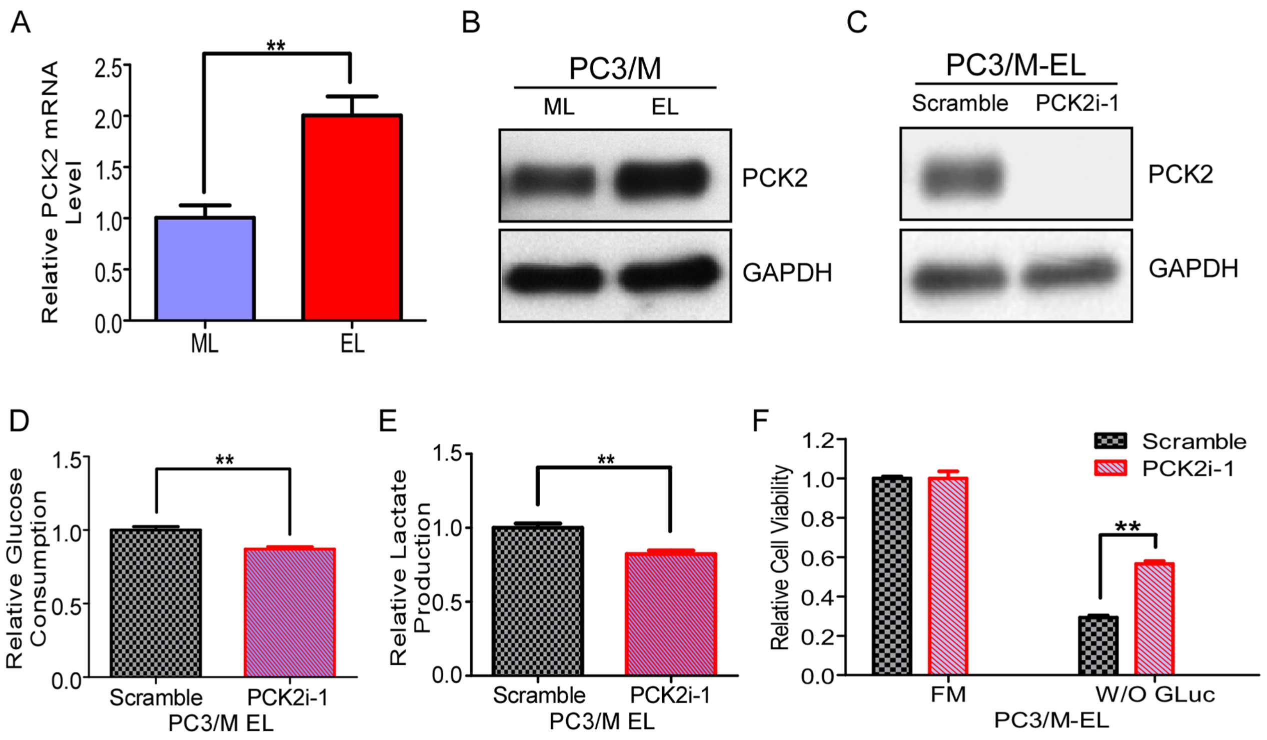

Supplementary Figure 3: High PCK2 expression in TIC-enriched prostate cancer cells is responsible for the enhanced glycolysis. (A and B) PCK2 expression in PC3/M-ML and PC3/M-EL clones detected by qPCR and immunoblotting. **(C)** PCK2 knockdown efficiency in PC3/M-EL cells, as detected by mmunoblotting. **(D)** Glucose consumption in the scramble control and PCK2-knockdown PC3/M-EL clone. **(E)** Lactate production by the scramble control and PCK2-knockdown PC3/M-EL clone. **(F)** Quantification of cell viability after two days of glucose deprivation (W/O Gluc). FM: full medium. **: $p < 0.01$.

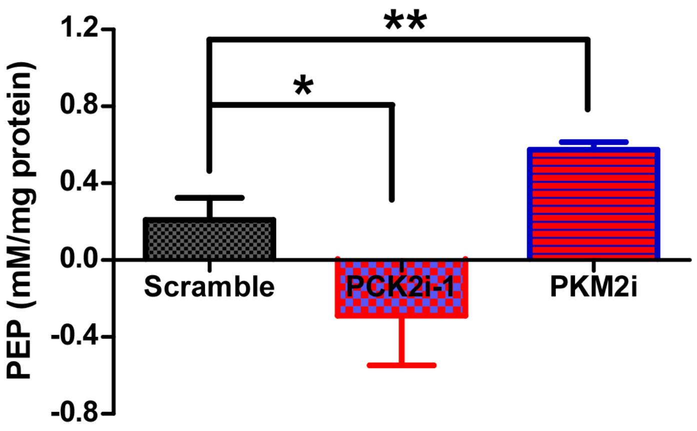

Supplementary Figure 4: PCK2 and PKM2 regulate PEP accumulation. Quantification of cellular PEP level in scramble control, and in PCK2- and PKM2-knockdown Du145-EL cells. *: $p < 0.05$; **: $p < 0.01$.

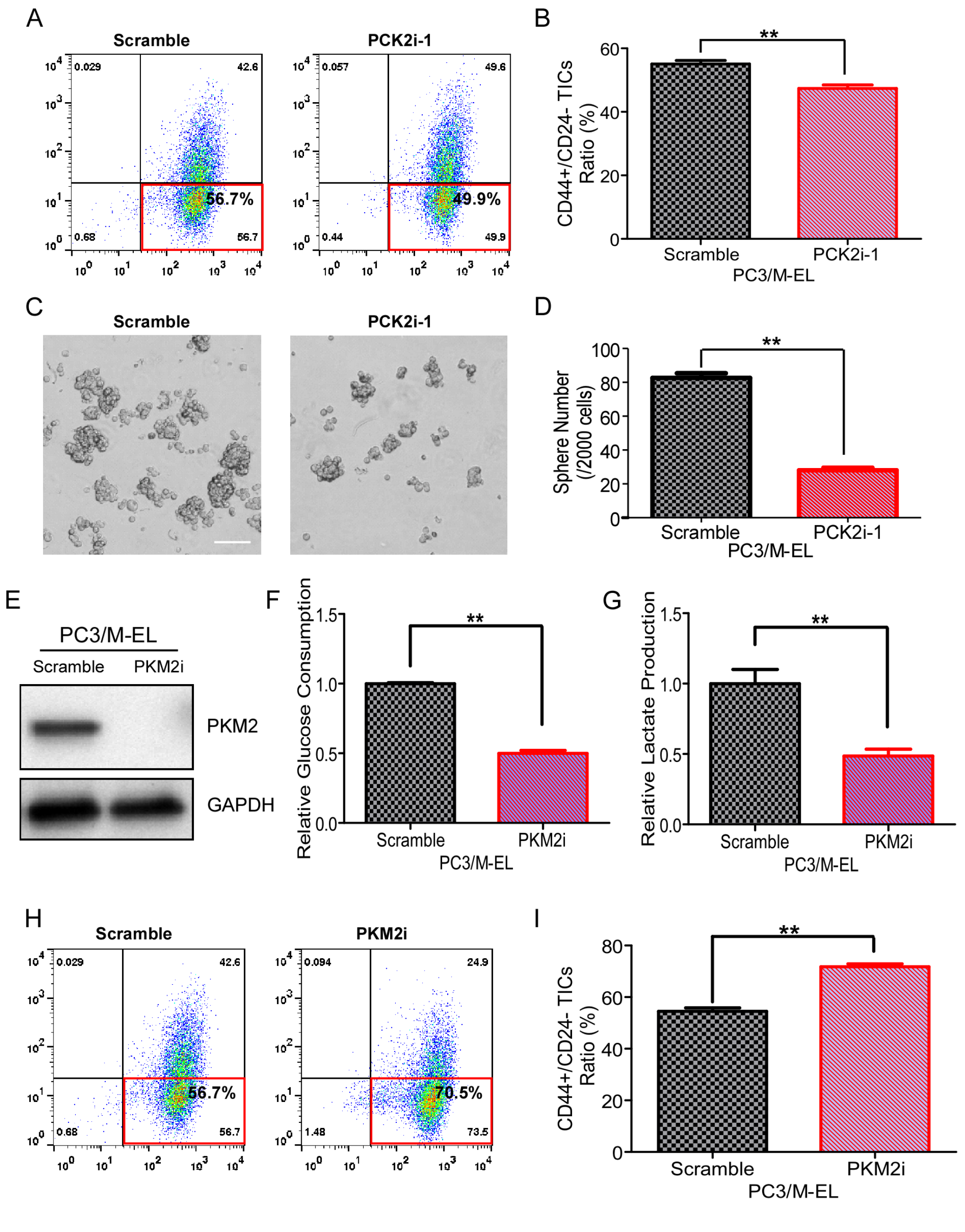

Supplementary Figure 5: PCK2 and PKM2 differentially regulate the TICs in PC3/M-EL cells. **(A)** Representative flow cytometry results of CD44+/CD24- TICs analyzed in scramble control and PCK2-knockdown PC3/M-EL cells. **(B)** Quantification of CD44+/CD24- TICs in scramble control and PCK2-knockdown PC3/M-EL cells. **(C)** Representative sphere formation results from scramble control and PCK2-knockdown PC3/M-EL cells. Scale bar: 100 μm. **(D)** Quantification of the spheres in scramble control and PCK2-knockdown PC3/M-EL cells. **(E)** PKM2 knockdown efficiency in PC3/M-EL cells, as detected by immunoblotting. **(F)** Glucose consumption detected in scramble control and PKM2-knockdown PC3/M-EL cells. **(G)** Lactate production detected in scramble control and PKM2-knockdown PC3/M-EL cells. **(H)** Representative flow cytometry results of CD44+/CD24- TICs in scramble control and PKM2-knockdown PC3/M-EL cells. **(I)** Quantification of the CD44+/CD24- TICs in scramble control and PKM2-knockdown PC3/M-EL cells. **: $p < 0.01$.

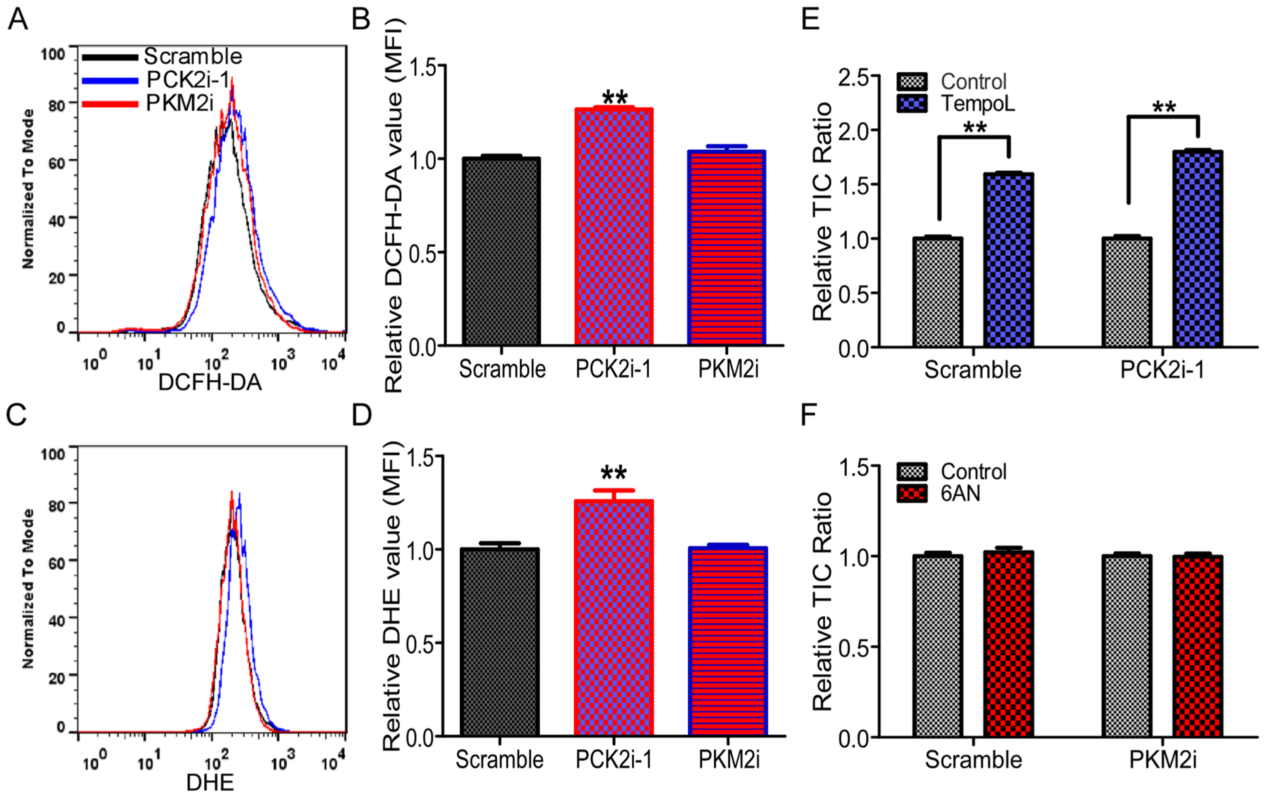

Supplementary Figure 6: PCK2 and PKM2 regulate cellular ROS in PC3/M-EL cells. **(A)** Representative flow cytometry results of the H_2O_2 level detected by DCFH-DA staining in scramble control, and in PCK2- and PKM2-knockdown PC3/M-EL cells. **(B)** Quantification of the H_2O_2 level in scramble control, and in PCK2- and PKM2-knockdown PC3/M-EL cells. MFI: mean fluorescence intensity, **: $p < 0.01$ (compared to scramble control). **(C)** Representative flow cytometry results of the $O2^{\cdot-}$ level detected by DHE staining in scramble control, and in PCK2- and PKM2-knockdown PC3/M-EL cells. **(D)** Quantification of the $O2^{\cdot-}$ level in scramble control, and in PCK2- and PKM2-knockdown PC3/M-EL cells. MFI: mean fluorescence intensity, **: $p < 0.01$(compared to scramble control). (E) Quantification of the CD44+/CD24- TICs in scramble control and PCK2-knockdown PC3/M-EL cells after being treated with 1 mM TempoL for two days. **: $p < 0.01$. **(F)** Quantification of the CD44+/CD24- TICs in scramble control and PKM2-knockdown PC3/M-EL cells after being treated with 10 µM 6-AN for two days.

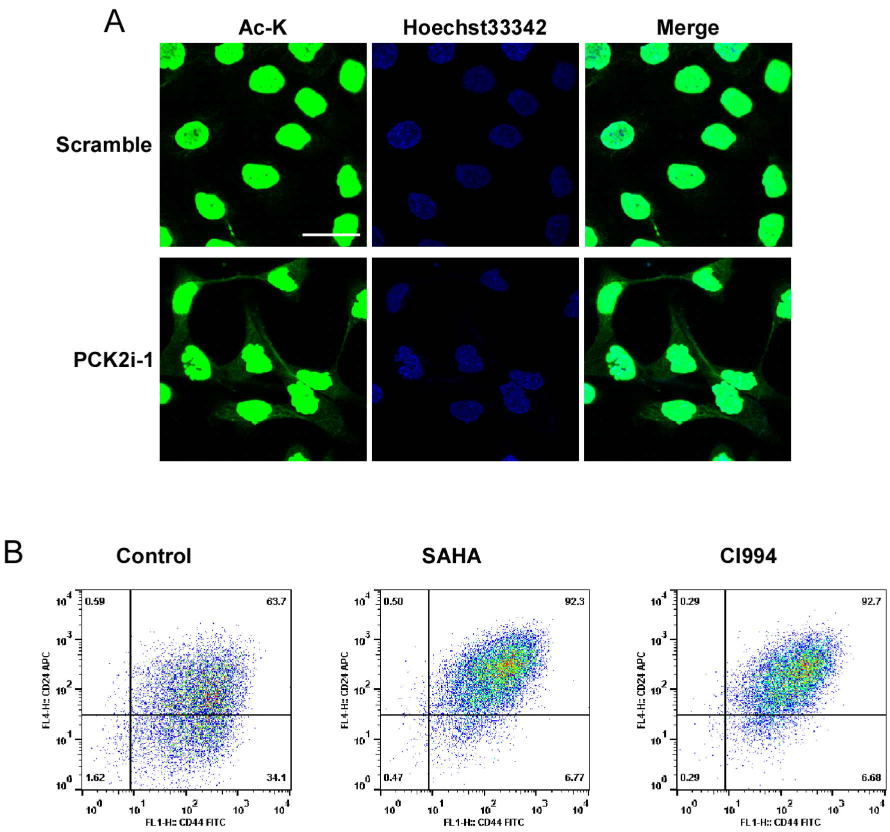

Supplementary Figure 7: PCK2 regulates cellular acetylation. (A) Protein acetylation in Du145-EL scramble and PCK2i-1 cells detected by immunofluorescence staining. Scale bar, 50 µm. **(B)** Representative flow cytometry results of CD44+/CD24- TICs in SAHA- and CI994-treated Du145-EL cells.
